# Tooth loss in young mice is associated with cognitive decline and femur-bone mineral density

**DOI:** 10.1007/s10266-024-01008-x

**Published:** 2024-10-04

**Authors:** Rie Hatakeyama, Hiroshi Oue, Miyuki Yokoi, Eri Ishida, Kazuhiro Tsuga

**Affiliations:** 1 Department of Advanced Prosthodontics, Hiroshima University Graduate School of Biomedical and Health Sciences, Hiroshima, Japan; 2 Department of Dentistry & Oral-Maxillofacial Surgery, Fujita Health University, Toyoake, Aichi Japan

**Keywords:** Tooth loss, Animal experiment, Behavioral test, Bone mineral density

## Abstract

Osteoporosis is a prevalent disease that is associated with increased hip fractures which cause significant decline in quality of life. Tooth loss affects systemic condition such as cognitive function through various mechanism, but the link between tooth loss and femoral bone mineral density is still uncertain. This study aims to investigate whether tooth loss in young mice affects memory function and femoral bone mineral density. Eight-week-old male C57BL/6 J mice were allocated randomly into the control group with sham operation and the tooth-loss group extracted all maxillary molar. Step-through passive avoidance test as cognitive function test, micro-CT analysis and western blotting analysis were performed after 1- and 2-month observation period. Step-through passive avoidance test revealed that the tooth-loss group in 2-month observation period impaired cognitive function. Additionally, micro-CT analysis revealed a significant decrease in both the length of the mandible and bone mineral density in the femur of the tooth-loss group compared to the control group. Claudin-5 level in the hippocampus, which is one of the tight junction markers in blood–brain-barrier, was significantly decreased in the tooth-loss group. The findings of our present study suggested that tooth loss impair cognitive function accompanied by reduced tight-junction marker, mandibular growth and bone mineral density of femur.

## Introduction

Osteoporosis is a prevalent condition worldwide, often defined operationally through bone mineral density (BMD) evaluation. The aging demographics globally are poised to significantly elevate osteoporosis rates among postmenopausal women [1]. Fragility fractures resulting from falls are a common clinical outcome of osteoporosis. Hip fracture represents the most severe complication of osteoporosis and is associated with elevated rates of morbidity, mortality, and healthcare expenses [2]. Given this large burden of morbidity in older adults, it is essential to address modifiable risk factors for fall, such as balance abnormalities, environmental factors and medication side effects, to prevent falls [3].

Recently, some studies have revealed that the residual teeth number is linked with hip fracture or femur BMD [4, 5]. Other researchers have also suggested that decreased residual teeth is a risk factor for difficulty balancing [6]. However, these reports were performed using a cross-sectional study design, and the mechanism of the association between tooth loss and bone fragility remains unclear.

Nutrition status change may significantly affect body composition or frailty [7]. Since tooth loss affects chewing ability and leads to poorer nutrition [8], we speculated that tooth loss affects bone fragility. The reduction in estrogen levels induces a persistent inflammatory condition, increasing the levels of pro-inflammatory cytokines like tumor necrosis factor-α (TNF-α) [9]. These factors influence the functioning of bone cells, thereby playing a role in the onset of osteoporosis [9, 10]. There are several shared risk factors (e.g., age, genetics, hormone change, smoking, calcium, and vitamin D) between osteoporosis and periodontitis [11]. A recent study has reported that tooth loss induces activation of glial cells in the hippocampus of Alzheimer’s disease model mice [12]. Our recent animal study have reported that tooth loss causes the change in blood–brain-barrier (BBB) composition [13]. It is widely acknowledged that inflammation affects BBB, including the role of pro-inflammatory cytokines and immune cell infiltration [14]. Also, impaired chewing function is a chronic stressor, resulting in elevated levels of circulating glucocorticoids, which induce cognitive deficits dependent on the hippocampus [15, 16]. Research indicates that psychologic stress can either inhibit or augment immune functions in both humans and experimental animals, contingent upon the particular stressor and the immune parameters under investigation [17]. Although the most of articles reported that tooth loss in experimental animals showed cognitive decline and pyramidal cell decrease in the hippocampus area [18], the pathological features, such as femur or mandibular bone deterioration, were not fully evaluated.

This study aims to investigate the impact of tooth loss on memory function and BMD in young mice.

## Materials and methods

**Fig. 1:**
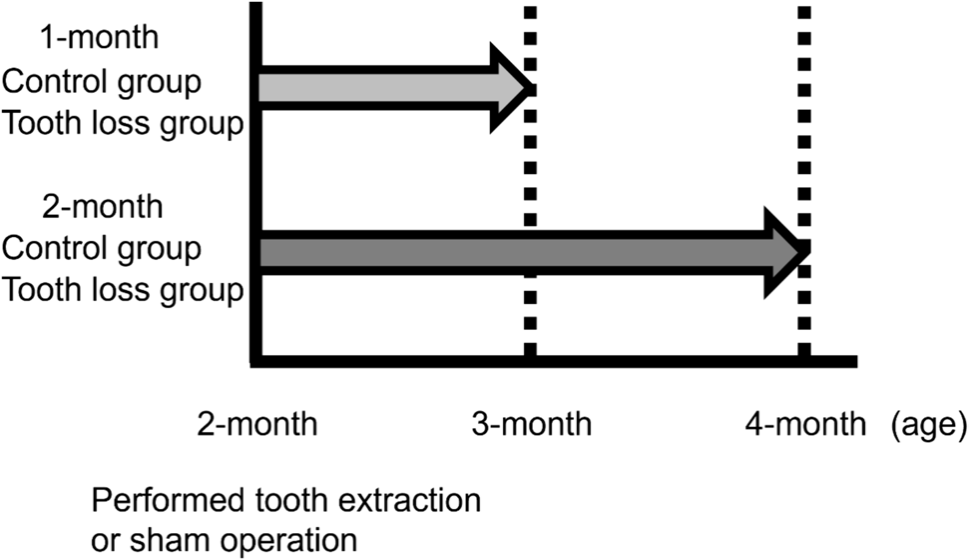
Study design. Mice were randomly undergoing tooth extraction or sham operation at 2 months of age. One month or 2 months later, the behavioral test or subsequent analysis were performed

Four-week-old C57 BL/6 J mice (male) were obtained from CLEA Japan, Inc. and raised in a 12-h light/dark cycle. These mice were allocated randomly into the tooth loss and control groups at 1-month or 2-month observation period, respectively (each group: *n* = 7). All maxillary molars were removed under intraperitoneal general anesthesia when the mice reached 8 weeks of age in the tooth loos group. Details regarding the anesthesia protocol can be found in our previous publications [13, 19]. Mice in the control group underwent anesthesia and a sham operation at the same age. All groups were provided with a standard diet pellet throughout the experimental period of one and two months from baseline. Body weights of the mice were measured at baseline, one month, and two months after baseline (Fig. 1). The animal study protocol received approval from Hiroshima University animal experiment ethics committee (Approval number: A20-129), and all animal experiments adhered to the ARRIVE guidelines.

### Learning and memory ability test

As outlined in prior research, a step-through passive avoidance test (PAT) was employed to examine learning and memory functions [20]. The device consisted of two compartments boxes with a light room and dark one separated by one door. In the first day, each mouse was initially introduced in the light room and permitted to investigate freely. After a 15 s delay, the mouse can move into the dark room by raising the door. The time taken until the mouse entirely entered the dark room was recorded as the latency time in the acquisition trial. Upon entry into the dark room, close the door that was open, and the mouse received an aversive stimulus (3 mA electric shock, 3 s) via the steel-rod grid. Subsequently, the mouse was reintroduced to its home cage. Latency time during the retention trial of the passive avoidance response was assessed the following day. After 24 h, the mouse was reintroduced to the light room for 15 s, and the latency time to enter the dark room was recorded without aversive stimulus. The latency time in acquisition trial and retention trial was measured with a maximum of 300 s per previous studies [19–21].

### Tissue preparation

After administering anesthesia, the mice underwent perfusion with saline. For western blotting (WB) analysis, their brains were extracted, flash-frozen in liquid nitrogen, and stored at − 80 °C. The hippocampi were homogenized in lysate buffer, which is a radioimmunoprecipitation assay (RIPA) buffer (Fisher Scientific) supplemented with a protease inhibitor (cOmplete^™^) and a phosphatase inhibitor (PhosSTOP^™^), followed by centrifugation at 12,000 rpm for 30 min at 4 °C. The obtained protein concentration was assessed utilizing a BCA protein assay kit from Thermo Fisher Scientific. The resulting supernatant was utilized for further analysis. Each sample was kept at − 80 °C for subsequent analysis. The mandibular and femur were resected and fixed in 4% paraformaldehyde (PFA) at 4 °C for subsequent μCT analysis.

### Micro-CT analysis

**Fig. 2:**
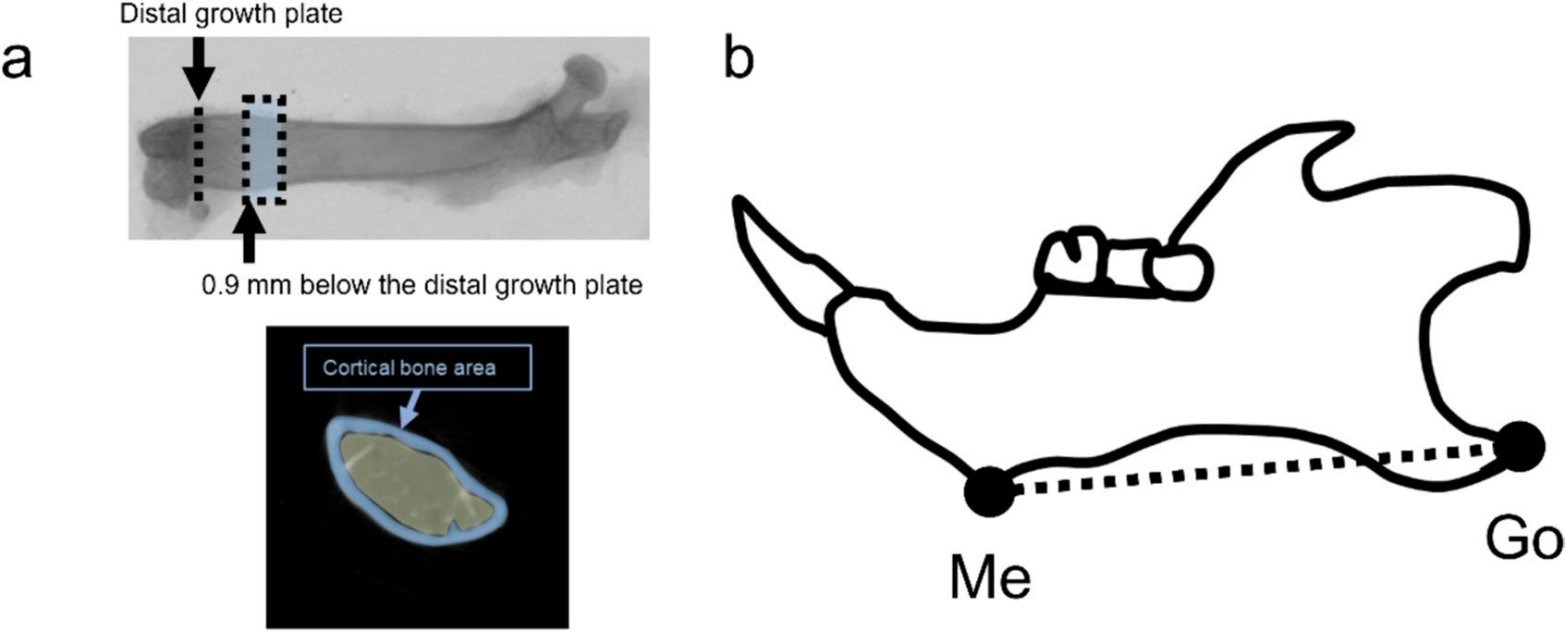
Landmarks used for micro-CT analysis. **a** The region of interest chosen for analysis of distal femur was positioned 0.9 mm below the growth plate to determine cortical BMD. **b** Me: point on the most inferior contour of the lower border of the mandible, adjacent to the incisors. Go: most posterior point of the angular process of the mandible

The mandibular and femur specimens underwent scanning with a SkyScan1176 scanner (Bruker) and were reconstructed using CTVOX software (Bruker) with a voxel size of 9 μm. CT-Analysis software (Bruker) was utilized to capture images to evaluate cortical bone BMD and cancellous bone Hounsfield unit (HU). The region of interest of cortical bone was positioned 0.9 mm below the growth plate and extended proximally 50 slices (Fig. 2a) [22]. The data were reconstructed using NRecon software (SkyScan). After data reconstruction, a morphologic assessment of the mandible was conducted using Dataviewer (Bruker, Kontich). Landmarks and measurement parameters for evaluating the mandibular through morphometric analysis were established as according to previous publications [23–25] (Fig. 2b). Three-dimensional measurements of the distance between the lowest contour of the mandible’s lower border, adjacent to the incisor (Me), and the rearmost point of the mandible’s angular process (Go) were performed.

### WB analysis

Equivalent amounts of protein were separated from homogenized tissue lysates, were subjected to 10% sodium dodecyl sulfate–polyacrylamide gel electrophoresis (SDS-PAGE) and transferred onto ethanol pre-soaked polyvinylidene fluoride (PVDF) membranes. Subsequently, the membranes were incubated with appropriately diluted primary antibodies overnight at 4 °C. After washing with Tris-buffered saline-Tween, the membranes were incubated with a blocking solution containing horseradish peroxidase-conjugated secondary antibodies (1:5000) at room temperature for 1 h. ChemiDoc XRS system (Bio-Rad) were used to visualize immunoreactive band. The primary antibodies used were anti-claudin 5 (1:1000, Invitrogen), anti-occludin (1:1000, Invitrogen), and anti-β-actin (1:1000, Abcam) serving as an internal control. Signal intensity of immunoreactive band was quantified using ImageJ program.

### Statistical analysis

Statistical analyses were performed using GraphPad Prism software. The obtained data were expressed as mean ± standard error and analyzed using Mann–Whitney test or Student’s t test. The data were considered statistically different at a value of *p* < 0.05.

## Results

### Body weight

**Fig. 3:**
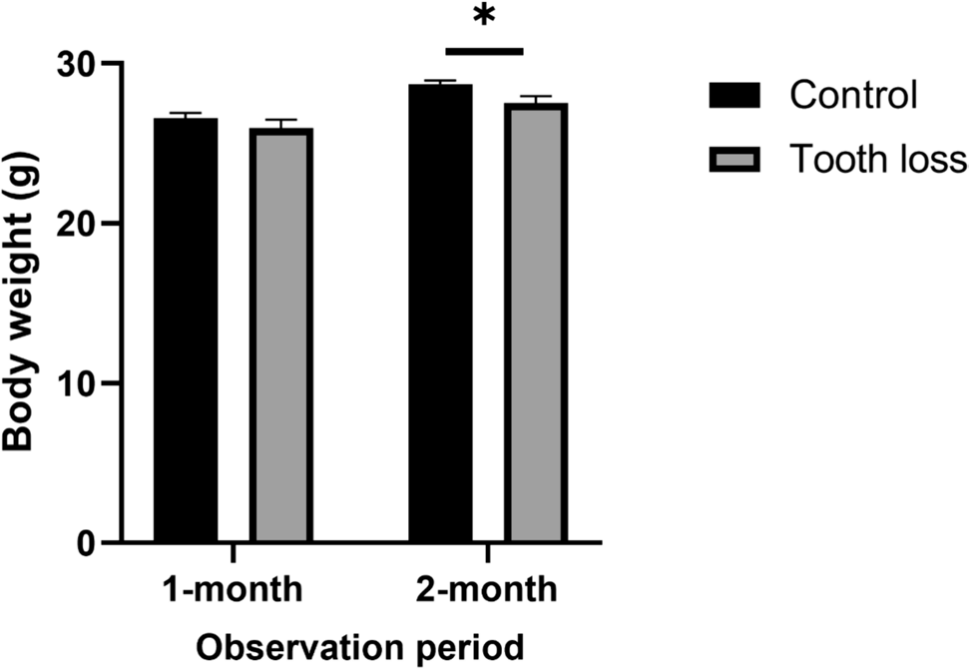
The tooth-loss group show reduced body weight at 2 month observation period. The bars expressed as mean ± S.E.M. *p < 0.05

The body weight results were as follows: 26.6 ± 0.3 g, 26.0 ± 0.5 g in the control group and the tooth-loss group in the 1-month observation period, 28.7 ± 0.2 g, 27.5 ± 0.4 g in the control group and the tooth-loss group in the 2-month observation period (Fig. 3). During the experiments, the average body weights of mice in both the control and tooth-loss groups marginally increased. No statistically significant difference was noted between the two groups in 1-month observation period, but significant difference was observed between the control and tooth-loss groups in 2-month period.

#### PAT

**Fig. 4:**
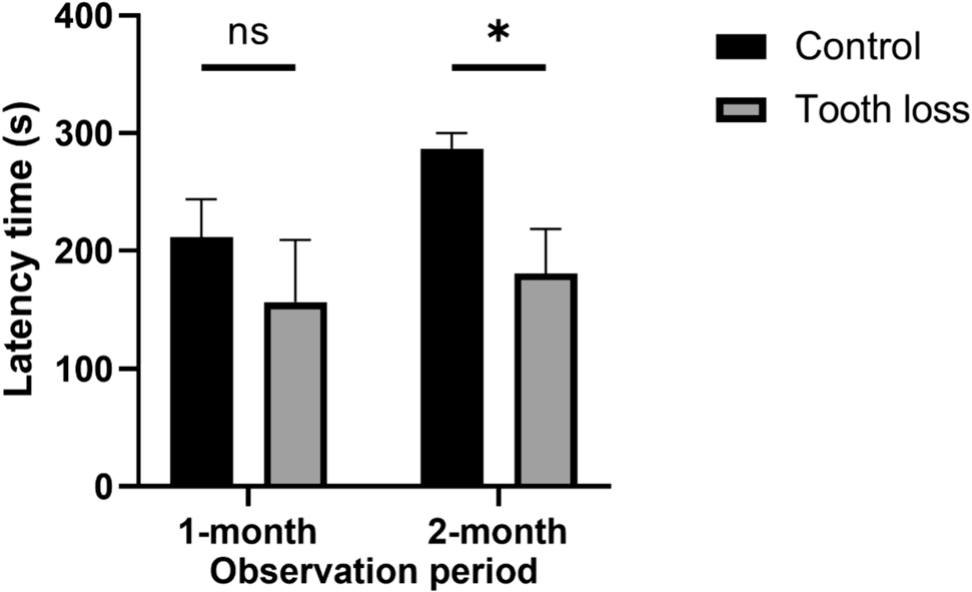
Tooth loss group show learning and memory deficit in the PAT. The bars indicate the mean latencies to enter the dark compartment in retention trial. At 1-month period, no significant differences observed in the mean retention latencies. In contrast at 2-month period, the mean retention latencies were significantly reduced in the tooth-loss group compared to the control group. Values are expressed as means ± S.E.M. *p < 0.05

In the acquisition trial, the latency time was not significantly different among the groups at 1- and 2-month observation period. Both the control group and tooth-loss group, the latency of retention trial was significantly longer than the latency of acquisition trial. It is noted that a significant reduction of the mean latencies during retention trial was observed in the tooth-loss group compared to the control group (*p* < 0.05) at the 2-month observation period (Fig. 4).

### Micro-CT analysis

**Fig. 5:**
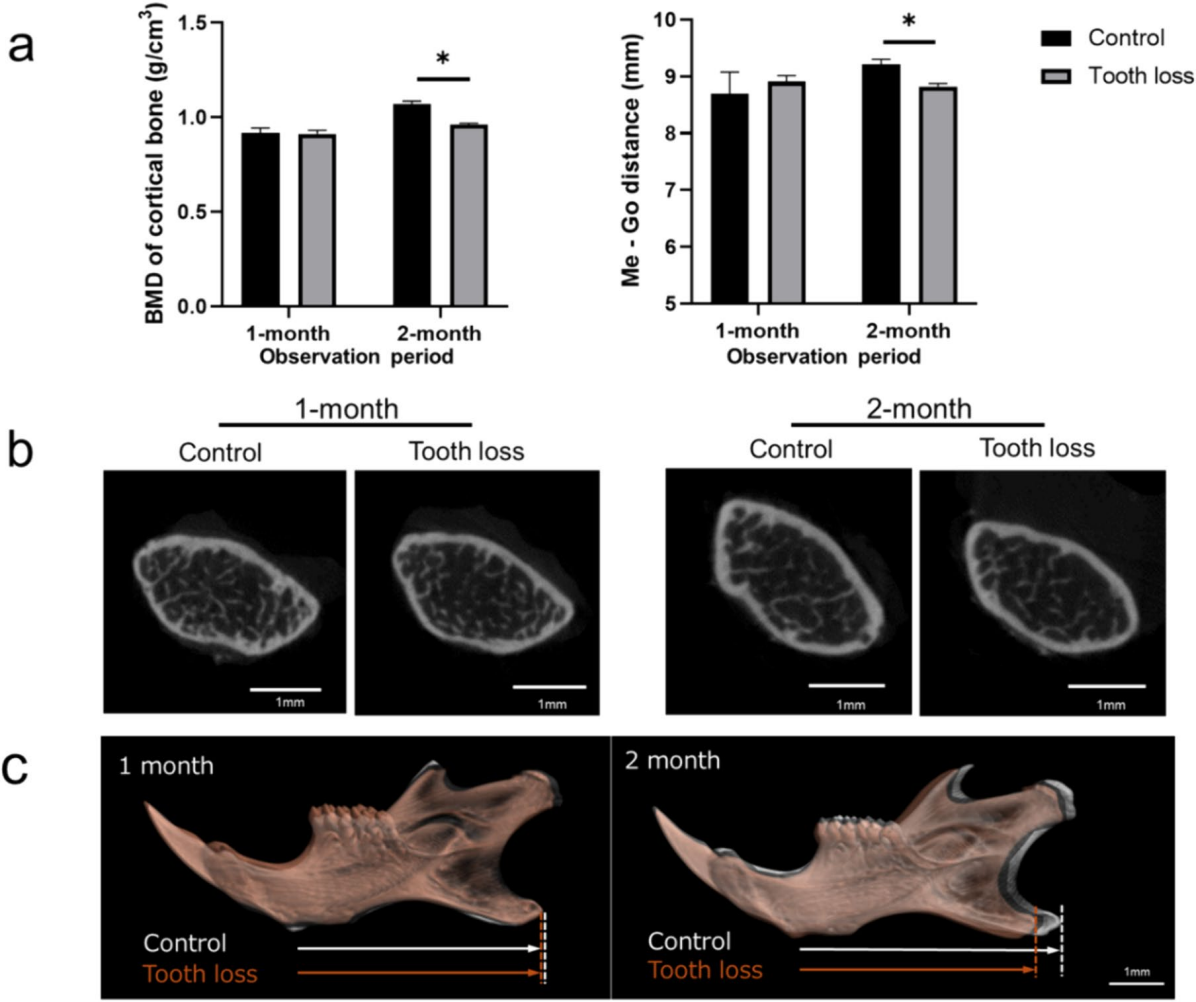
micro-CT analysis. **a** The Me – Go and BMD were significantly lower in the tooth-loss group than the control group at 2-month period, but not at 1-month period. The bars expressed as mean ± S.E.M. *p < 0.05. **b** Representative micro-CT image of femoral cortical area. **c** Representative overly of micro-CT image of mandibular

Throughout a 1-month observation period, no discernible differences in BMD were observed at the distal end of the femur (Fig. 5a). However, over the 2-month observation period, a decrease in femur BMD was noted in the tooth-loss group compared to the control group. In the 2-month observation period, the length of the mandible (Me-Go) was notably shorter in the tooth-loss group compared to the control group, whereas no significant difference was observed during the 1-month observation period (Fig. 5b, c).

### WB analysis

**Fig. 6:**
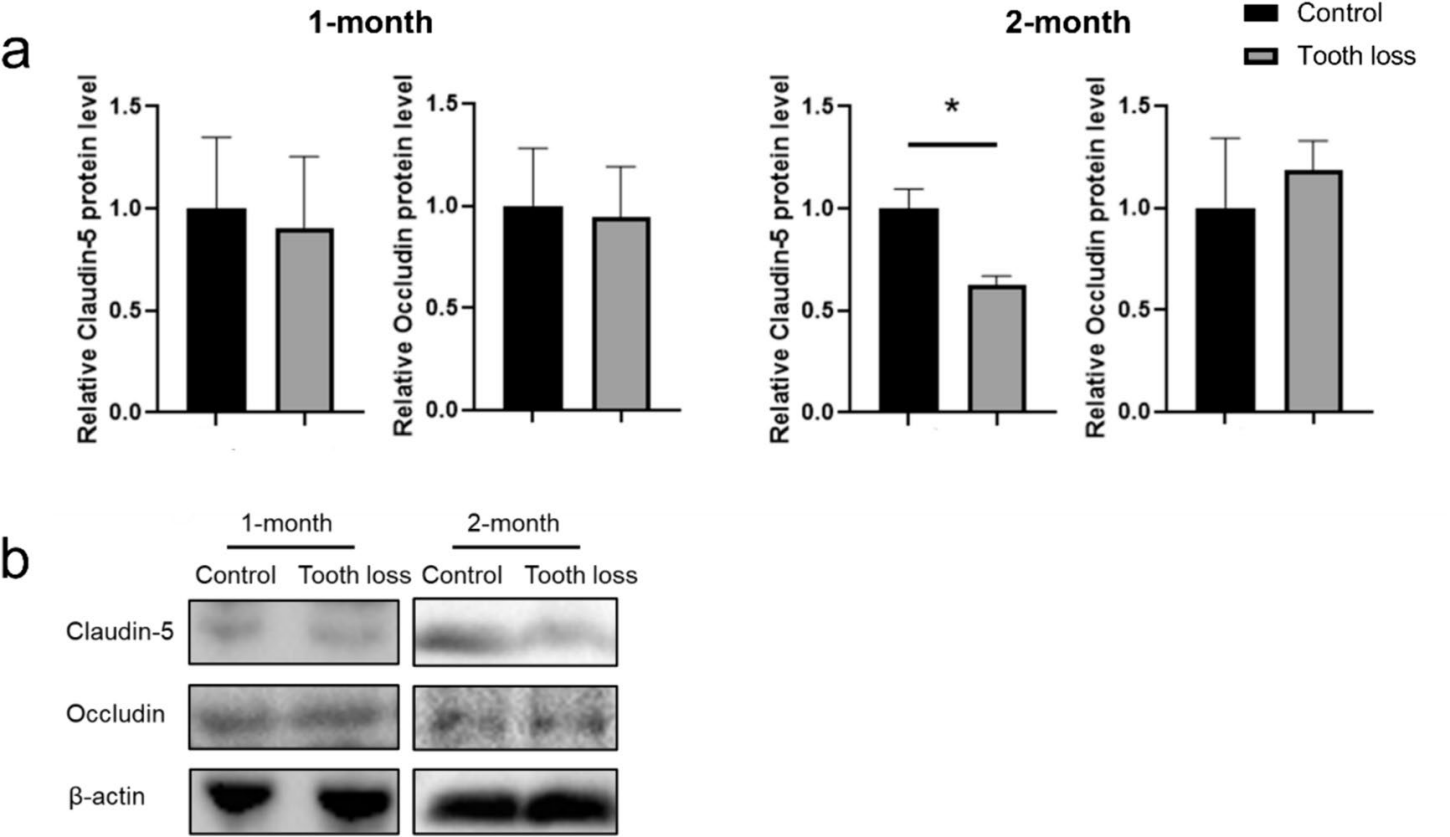
WB analysis. **a** Tooth loss decreases claudin-5 level in the hippocampus at 2-month period, but not at 1-month period. The bars expressed as mean ± S.E.M. * *p* < 0.05. **b** Representative western blot image of claudin-5, occluding and β-actin

To investigate the influence of tooth loss on tight junction levels, tight junction markers were used for WB. The protein level of claudin-5 and occludin revealed no significant difference between the control and tooth-loss groups over the 1-month observation period (Fig. 6). However, the claudin-5 level in the hippocampus of the tooth-loss group was found to have a significant reduction compared to the control group in the 2-month observation period. Conversely, occluding protein levels did not exhibit significant differences between the groups at 1- and 2-month observation period.

## Discussion

The current study showed that experimental maxillary molar loss in young mice resulted in memory deficits, mandibular growth deterioration, and reduced BMD in the femur over 2-month observation period, but not 1-month observation period.

Body weight is directly associated with BMD [26]. Some previous tooth loss experimental studies showed that slight weight loss in animals was observed [27, 28]. In contrast, the other study showed that tooth loss did not cause body weight loss during the observation period [29, 30]. The length of the mandibular was decreased in the tooth-loss group. Throughout the growth period, the jawbone experiences specific forces exerted by surrounding tissues, such as the teeth and muscles of the mouth, including masticatory. As increased mastication is reported to regulate jawbone formation [31], it is indicated that tooth loss may impair normal mastication and normal mandibular growth. In this study, body weight is significantly different in the 2-month observation period. However, the tooth-loss group experienced a decrease in their standard body weight of no more than 5%. Therefore, we excluded the possibility of malnutrition in the tooth-loss group.

In the context of PAT, the latency time in the retention trial decreased in the tooth-loss group compared with that in the control group. Prolonged latency in the retention trial typically results from the recognition of a fearful memory, such as aversive stimulus encountered during the acquisition trial. This outcome implies that tooth loss may expedite memory deficits. The fact that tooth loss affects systemic health through various mechanisms is now widely accepted [32]. Tooth loss resulting from dental caries and periodontitis has the potential to initiate or worsen conditions such as diabetes, endocarditis, other inflammatory diseases, and AD [33]. According to previous reports, tooth loss provokes chronic stress in mice, triggering hypothalamic–pituitary–adrenal (HPA) axis activation and resulting in elevated corticosterone levels [34]. The hippocampus is vulnerable to stress and aging, being among the initial brain areas that undergo structural and functional alterations due to stress [35]. Adrenal glands release glucocorticoids, such as corticosterone, in response to stressors, which are recognized as immune system regulators [36]. Glucocorticoids are reported to suppress inflammation [37], whereas other studies showed that glucocorticoids may have little effect in controlling inflammation [38]. However, another article mentioned that glucocorticoids can influence bone remodeling in several ways, including the increase of pro-inflammatory cytokines such as IL-1β and IL-6, and at any stage of the remodeling cycle [39]. Taken together, tooth loss seems to trigger an increasing inflammatory response to chronic stressors through the production of glucocorticoids.

Neurodegenerative diseases and aging have different pathogenetic mechanisms, but all share the hallmark of chronic neuroinflammation [40]. Our previous study demonstrated that the long-term effect of tooth loss caused a decrease in claudin-5 levels, which regulates an infiltration of peripheral immune cells into the central nervous system in BBB [13]. Inflammatory conditions can contribute to BBB disruption, where excessive brain immune response disturbs endothelial barrier integrity in neurodegenerative diseases [41, 42]. Claudin family are an integral protein that regulates BBB permeability [43]. We considered that tooth loss the impact of tooth loss is limited to disrupt tight junction. However, this result suggests that tooth loss may accelerate the pathology of the neurodegenerative disease process via neuroinflammation. Notably, individuals diagnosed with neurodegenerative diseases face a heightened susceptibility to metabolic bone disorders in comparison to controls of similar age [44]. Chronic mild inflammation may pose a risk factor for reduced BMD and fracture in older populations [45, 46]. Previously, it has been contended that osteoporosis influences alveolar bone resorption and tooth loss [47]. Although our results demonstrated that the BMD of the femur in the tooth-loss group decreased, the direct involvement of tooth loss in decreased BMD remains uncertain.

There are some limitations of this study. Although the changes in BMD and memory function were observed by experimental tooth loss in this study, the causation is still unclear. Also, whether tooth loss is directly implicated in inflammatory response remain still unclear because we did not assess the inflammatory response in the brain or femur. Previous study suggested that tooth loss increased glial activation in mice brain [12]. One possible mechanism is that tooth loss causes reduced afferent sensory input or increased glucocorticoids, which are associated with an inflammatory response and a subsequent BMD decrease. We only assessed biochemical analysis by WB analysis, because the interrelationship between tooth loss and cognitive function is so complicated as previous report showed schematic figure [18]. Further study is needed to clarify how tooth loss affects neuroinflammation or bone metabolism by advanced techniques such as single-cell RNA sequencing or OMICs analysis.

In conclusion, this study demonstrated that tooth loss impairs cognitive function and femur BMD in young mice, accompanied by a change of tight junction level in brain. As untreated tooth loss may be a risk factor for cognitive impairment and osteoporosis, adequate dental treatment, including prosthodontic treatment or masticatory improvement, is essential.

## Data Availability

The corresponding authors will make the data available on reasonable request.
